# Survival Comparisons of Hepatic Arterial Infusion Chemotherapy With mFOLFOX and Transarterial Chemoembolization in Patients With Unresectable Intrahepatic Cholangiocarcinoma

**DOI:** 10.3389/fonc.2021.611118

**Published:** 2021-04-01

**Authors:** Zhiyuan Cai, Chaobin He, Chongyu Zhao, Xiaojun Lin

**Affiliations:** ^1^ Department of Pancreatobiliary Surgery, State Key Laboratory of Oncology in South China, Collaborative Innovation Center for Cancer Medicine, Sun Yat-sen University Cancer Center, Guangzhou, China; ^2^ Guangdong Provincial Engineering Research Center of Molecular Imaging, The Fifth Affiliated Hospital of Sun Yat-sen University, Zhuhai, China

**Keywords:** intrahepatic cholangiocarcinoma, transarterial chemoembolization, hepatic arterial infusion chemotherapy, overall survival, only intrahepatic progression-free survival

## Abstract

**Background:**

Intrahepatic cholangiocarcinoma (ICC) has a poor prognosis and 40%-60% of patients present with advanced disease at the time of diagnosis. Transarterial chemoembolization (TACE) and hepatic arterial infusion chemotherapy (HAIC) have recently been used in unresectable ICC. The aim of this study was to compare the survival differences of unresectable ICC patients after TACE and HAIC treatment.

**Methods:**

Between March 2011 and October 2019, a total of 126 patients with unresectable ICC, as evident from biopsies and imaging, and who had received TACE or HAIC were enrolled in this study. Baseline characteristics and survival differences were compared between the TACE and HAIC treatment groups.

**Results:**

ICC Patients had significantly higher survival rates after the HAIC treatment, compared with those after TACE treatment [1-year overall survival (OS) rates: 60.2% vs. 42.9%, 2-year OS rates: 38.7% vs. 29.4%, P=0.028; 1-year progression-free survival (PFS) rates: 15.0% vs. 20.0%, 2-year PFS rates: 0% vs. 0%, P=0.641; 1-year only intrahepatic PFS (OIPFS) rates: 35.0% vs. 24.4%, 2-year OIPFS rates: 13.1% vs. 14.6%, P = 0.026]. Multivariate Cox regression analysis showed that HAIC was a significant and independent factor for OS and OIPFS in the study cohort.

**Conclusions:**

HAIC is superior to TACE for treatment of unresectable ICC. A new tumor response evaluation procedure for HAIC treatment in unresectable ICC patients is needed to provide better therapeutic strategies. A randomized clinical trial comparing the survival benefits of HAIC and TACE is therefore being considered.

## Introduction

Intrahepatic cholangiocarcinomas (ICC) arising from epithelial cells of the intrahepatic bile ducts account for 10%-20% of newly diagnosed hepatic malignancies and are increasing in incidence (1, 2). Overall, 3-year and 5-year survival rates of ICC are only 31% and 18%, respectively (3). Surgical resection is the only potentially curative therapy. However, in the absence of specific clinical symptoms, 40%-60% of patients present with advanced disease at the time of diagnosis. Due to multiple intrahepatic lesions, local infiltration and lymph node and distant metastases, many patients are unable to undergo operative procedures (4, 5). The prognosis for patients with unresectable cholangiocarcinoma is very poor, with 2.5-7.5 months of median survival time in the absence of treatment (6).

Although previous study showed that GEMOX chemotherapy was the recommended therapy for cholangiocarcinoma patients, while the response rate was 21.4%, and the survival benefits were limited, with the median progression-free survival (PFS) and overall survival (OS) time of 2.5 and 14.5 months, respectively (7). Similarly, in the ARC-02 trial, the OS of patients with advanced biliary cancer was only 11.7 months after cisplatin plus gemcitabine chemotherapy (8). The FOLFOX regimen may be a novel option in the palliative treatment of advanced cholangiocarcinoma, demonstrating a disease control rate of 56% and a median OS time of 9.5 months (9). Although systemic chemotherapy is the first-line adjuvant therapy for patients with unresectable ICC, its effects are often limited (10). Transarterial chemoembolization (TACE), concentrating chemotherapeutics on the tumor while blocking tumor-feeding arteries, is an important therapeutic procedure in patients with unresectable ICC. TACE is a safe method that prolongs overall survival in these patients (11, 12). Hepatic arterial infusion chemotherapy (HAIC)-injecting chemotherapeutic agents into the hepatic artery without embolization-reduces the systemic side effects seen with systemic chemotherapy (13). Previous studies have illustrated that HAIC is a promising option for advanced ICC and has shown higher tumor control rates than systemic chemotherapy (14). Chemotherapy with hepatic intraarterial epirubicin and cisplatin combined with systemic 5-fluorouracil (5-FU) was used in patients with unresectable ICC, and the objective response rate and median survival time were 36% and 15.4 months, respectively (15). For patients with unresectable ICC, TACE and HAIC are both important treatments and show reasonable outcomes of tumor response and overall survival (16). However, no trials comparing TACE and HAIC outcomes have been performed.

The aim of the present study was to compare the clinical response and survival differences after either TACE or HAIC in patients with unresectable ICC. The study represented a retrospective review of a consecutive series of patients with unresectable ICC treated with TACE and HAIC over a nine-year period.

## Method

### Patient Characteristics

All primary unresectable ICC patients who were initially treated with TACE or HAIC between March 2011 and October 2019 at Sun Yat-sen University Cancer Center were identified. A total of 126 patients were included, based on the following inclusion criteria: (1) ICC confirmed by clinical and histopathological evidence; (2) patients who was not suitable for radical surgery because of advanced disease status; (3) patients who were 18 years or older; (4) patients with Child-Pugh A and B cirrhosis; (5) patients with completed follow-up data; and (6) patients who gave informed consent voluntarily. Patients were excluded based on the following exclusion criteria: (1) patients who had contraindications to TACE and HAIC; and (2) patients with a history of second primary malignant tumors.

### Data Collection

All clinical data for diagnosis were obtained from medical records filed at Sun Yat-sen University Cancer Center. The following data were collected and analyzed: age, gender, tumor size, vascular invasion of tumor, lymph node (LN) metastasis, distant metastasis, tumor-node-metastasis (TNM) stage, white blood cell (WBC) count, hemoglobin (HGB), platelet (PLT) count, serum albumin levels (ALB), alanine transaminase (ALT), alkaline phosphatase (ALP), aspartate aminotransferase (AST), glutamyl transpeptidase (GGT), indirect bilirubin (IBIL), total bilirubin (TBIL), C-reactive protein (CRP), alpha fetoprotein (AFP), carcinoembryonic antigen (CEA), carbohydrate antigen 19-9 (CA19-9), protein induced by Vitamin K absence II (PIVKA-II), Hepatitis B virus surface antigen (HBsAg) and treatment with TACE and HAIC. The study was followed up until October 30, 2019. By the end of the follow-up, 40 patients had died and 86 patients survived.

OS was defined as the interval from the date of the first TACE or HAIC treatment to death or the last follow-up. PFS was defined as the interval from the date of the first TACE or HAIC treatment to the date when tumor progression was diagnosed or the last follow-up. Only intrahepatic progression-free survival (OIPFS) was defined as the interval from the date of first TACE or HAIC treatment to the date when only intrahepatic tumor progression was diagnosed or the last follow-up regardless of whether it was accompanied by extrahepatic metastasis. On the basis of the Response Evaluation Criteria in Solid Tumors (RECIST) (17), tumor responses were evaluated by two hepatobiliary surgeons. All objective tumor responses were confirmed at least 4 weeks after the first treatment.

### Transarterial Chemoembolization

The Seldinger technique was used to intubate the femoral artery up to the proper hepatic artery or its branches. Superselective catheterization up to the tumour blood supply artery was carried out after confirming the location, number, size and vascular supply of the tumors by angiography. Chemotherapeutic agents were infused through the tumor blood supply artery, and embolization was performed with iodized oil. The amount of iodized oil varied from 3 to 25 ml, and individualized treatment was carried out according to the location, size and number of tumors. In the procedure of TACE, the perfusion drugs were 50 mg of epirubicin, 6 mg of mitomycin and 300 mg of carboplatin.

### Hepatic Arterial Infusion Chemotherapy

A microcatheter was selectively placed into the tumor blood supply artery. If necessary, the gastroduodenal artery was occluded. The microcatheter was then connected to the artery infusion pump to administer the following treatment (mFOLFOX): 85 mg/m^2^ OXA intra-arterial infusion on day 1, 400 mg/m^2^ LV intra-arterial infusion on day 1, and 400mg/m^2^ 5-FU bolus infusion on day 1, and 2400 mg/m^2^ 5-FU continuously infused over 46 h (18). Patients received six to eight courses (a 21-day cycle regimen) of treatment, and the therapy was discontinued if it was not well-tolerated for another course of HAIC.

### Statistical Analysis

All variables were divided into categorical variables and were compared using the chi-square test. The OS, PFS and OIPFS curves were analyzed by the Kaplan-Meier method, and differences between the groups were compared using the results of the log-rank test. Multivariate analysis was performed with the Cox regression model for variables that were significant in the univariate analysis, and a P value < 0.05 was deemed significant. All statistical analyses were performed by using the Statistical Package for Social Sciences version 22.0 (SPSS Inc., Chicago, IL, USA).

## Results

### Patient Characteristics

The clinical and imaging data are shown in **Table 1**. A total of 126 unresectable ICC patients were included in this study, including 69 patients receiving TACE treatment and 57 patients receiving HAIC treatment. The thresholds of the clinical variables were defined as their cutoff values. According to our previous results (19), compared with 35 U/ml, 200 U/ml was superior for survival prediction as a cutoff value of CA19-9. Therefore, 200 U/ml was used as a cutoff value for CA19-9. The CA19-9 effect was divided into three categories: negative before treatment, and not declining after treatment if positive before treatment and no declining after treatment if positive before treatment. All clinical variables were balanced between the HAIC and TACE groups.

**Table 1:** Comparisons of clinical characteristics of patients.

Characteristic	HAIC (n = 57)	TACE (n = 69)	P
gender			
male	42	53	0.685
female	15	16	
age			
≤60	42	48	
>60	15	21	0.610
Tumor size			
≤5	5	5	0.753
>5	52	64	
Vascular Invasion			
Absence	28	34	0.986
Presence	29	35	
LN metastasis			
Absence	25	22	0.167
Presence	32	47	
Distant metastasis			
Absence	37	43	0.763
Presence	20	26	
TNM stage			
II	22	15	0.096
IIIb	15	28	
IV	20	26	
WBC (E+09)			
≤10	40	50	0.777
>10	17	19	
HGB (g/L)			
≤120	14	18	0.845
>120	43	51	
PLT (E+09)			
≤300	45	56	0.757
>300	12	13	
ALB (g/L)			
≤40	24	34	0.422
>40	33	35	
ALT (U/L)			
≤50	43	56	0.436
>50	14	13	
AST (U/L)			
≤40	27	39	0.306
>40	30	30	
ALP (U/L)			
≤125	24	30	0.877
>125	33	39	
GGT (U/L)			
≤60	6	12	0.273
>60	51	57	
TBIL (umol/L)			
≤20.5	50	55	0.230
>20.5	7	14	
IBIL (umol/L)			
≤15	54	64	0.650
>15	3	5	
CRP (ng/L)			
≤3	7	7	0.704
>3	50	62	
AFP (ng/ml)			
≤25	38	42	0.501
>25	19	27	
CEA (ng/mL)			
≤5	34	46	0.415
>5	23	23	
CA19-9 (U/ml)			
≤35	17	24	0.517
>35	40	44	
HBsAg			
negative	24	30	0.831
positive	32	37	
CA19-9 (U/ml)			
≤200	38	40	0.317
>200	19	29	
CA19-9effect			
Negative before treatment	15	14	0.720
Decline after treatment	24	15	
No decline after treatment	11	8	
PIVKA-II(mAU/ml)			
≤40	34	20	0.698
>40	17	12	

### OS, PFS, and OIPFS in All Patients

**Figure 1:**
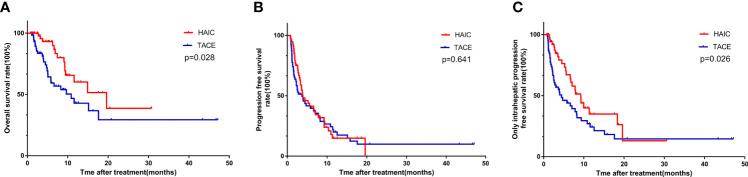
The Kaplan-Meier survival curves of overall survival **(A)** progression free survival **(B)** and only intrahepatic progression free survival **(C)** stratified by treatment strategies for patients with unresectable ICC.

The median follow-up time was 8.4 months (range 0.8–47.2 months) for the entire research cohort. During follow-up, 14 patients (24.6%) in the HAIC group and 26 patients (37.7%) in the TACE group died (P = 0.115). The median OS times in the HAIC and TACE groups were 19.6 and 10.8 months, respectively, while the 1-year and 2-year OS rates in the HAIC and TACE groups were 60.1% and 38.6% and 42.9% and 29.4%, respectively (P = 0.028, **Figure 1A**). Patients in the HAIC group had significantly longer OS times than those in the TACE group. There were 40 (70.2%) patients in the HAIC group and 48 patients (69.6%) in the TACE group had tumors progressed during the follow-up period (P = 0.941). The median PFS times in the HAIC and TACE groups were 3.9 and 3.7 months, respectively (P = 0.641, **Figure 1B**). There was no obvious difference in the PFS of patients between the HAIC group and the TACE group. After tumor responses were estimated according to RECIST, 24 (42.1%) patients in the HAIC group and 41 (59.4%) patients in the TACE group had intrahepatic tumor progression (P = 0.053). The median OIPFS in the HAIC and TACE groups were 9.2 and 4.4 months, respectively, while the 1-year and 2-year OIPFS rates in the two groups were 35.0% and 24.4% and 13.1% and 14.6%, respectively (P = 0.026, **Figure 1C**). Patients in the HAIC group had significantly longer OIPFS times than patients in the TACE group.

**Figure 2:**
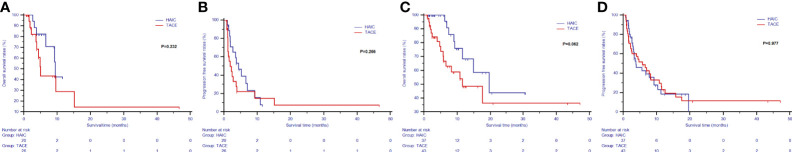
The Kaplan-Meier survival curves of overall survival **(A, C)** progression free survival **(B, D)** stratified by treatment strategies for ICC patients with and without metastasis, respectively.

The prognostic analysis of all clinical variables was conducted using Cox regression analysis. Univariate analysis for OS revealed that treatment [TACE vs. HAIC, hazard ratio (HR) = 2.045; 95% CI, 1.067-3.920; P = 0.031] and distant metastasis (presence vs. absence, HR = 1.975, 95% CI, 1.039-3.765; P = 0.038) were related to OS (**Table 2**). After multivariate analysis, treatment (TACE vs. HAIC, HR = 2041; 95% CI, 1.065-3.913; P = 0.032) was the independent prognostic factor for OS. Univariate analysis for OIPFS showed that treatment (TACE vs. HAIC, HR = 1.758; 95% CI, 1.061-2.913; P = 0.029), TNM stage (II vs. IIIb vs. IV, HR = 1.532, 95% CI, 1.114-2.107, P = 0.009) and distant metastasis (Presence vs. Absence, HR=1.975, 95% CI, 1.039-3.765, P=0.038) were related to OS. After multivariate analysis, treatment (TACE vs. HAIC, HR = 1.862; 95% CI, 1.098-3.159; P = 0.021) was the independent prognostic factor for OIPFS (**Table 2**). In patients with distant metastases, there were 20 patients in HAIC group and 26 patients in TACE group. It was shown that no significant differences in OS (P = 0.232, **Figure 2A**) and PFS (P = 0.266, **Figure 2B**) were observed in these two groups. Furthermore, in patients without distant metastases, no significant differences in OS (P = 0.062, **Figure 2C**) and PFS (P = 0.977, **Figure 2D**) were observed between the TCAE group (37 patients) and HAIC group (43 patients).

**Table 2:** Univariate and multivariate analyses of survival in patients.

Characteristic		Overall survival						Only intrahepatic progression-free survival					
		Univariate analysis			Multivariate analysis			Univariate analysis			Multivariate analysis		
		HR	95%CI	P	HR	95%CI	P	HR	95%CI	P	HR	95%CI	P
treatment	HAIC/TACE	2.045	1.067-3.920	0.031	2.047	1.067-3.928	0.031	1.758	1.061-2.913	0.029	1.862	1.098-3.159	0.021
gender	male/female	1.612	0.795-3.268	0.185				1.547	0899-2.662	0.115			
age	≤60/>60	0.951	0.474-1.909	0.888				0.894	0.514-1.557	0.693			
Tumor size	≤5/>5	0.856	0.304-2.410	0.769				0.949	0.409-2.201	0.903			
Vascular invasion	Absence/Presence	1.258	0.672-2.357	0.473				0.905	0.555-1.475	0.688			
LN metastasis	Absence/Presence	1.617	0.821-3.184	0.164				1.652	0.973-2.802	0.063			
Distant metastasis	Absence/Presence	1.975	1.039-3.765	0.038	1.972	1.039-3.742	0.038	2.059	1.249-3.394	0.005	2.312	0.801-6.678	0.121
TNM stage	II/IIIb/IV	1.429	0.948-2.155	0.089				1.532	1.114-2.107	0.009	0.946	0.493-1.815	0.868
WBC (E+09)	≤10/>10	0.673	0.317-1.429	0.303				1.23	0.727-2.082	0.441			
HGB (g/L)	≤120/>120	1.116	0.513-2.428	0.782				1.041	0.584-1.856	0.892			
PLT (E+09)	≤300/>300	0.925	0.407-2.103	0.853				1.143	0.620-2.108	0.668			
ALB (g/L)	≤40/>40	0.872	0.464-1.638	0.67				0.823	0.504-1.343	0.435			
ALT(U/L)	≤50/>50	1.19	0.593-2.387	0.625				0.879	0.485-1.593	0.671			
AST (U/L)	≤40/>40	1.652	0.875-3.119	0.121				0.847	0.518-1.383	0.506			
ALP (U/L)	≤125/>125	1.264	0.670-2.383	0.469				0.969	0.592-1.586	0.901			
GGT (U/L)	≤60/>60	1.521	0.539-4.291	0.428				1.05	0.518-2.130	0.892			
TBIL (umol/L)	≤20.5/>20.5	1.065	0.447-2.539	0.887				0.757	0.361-1.588	0.462			
IBIL (umol/L)	≤15/>15	0.98	0.132-7.258	0.984				1.347	0.417-4.348	0.618			
CRP (ng/L)	≤3/>3	1.731	0.614-4.879	0.3				1.648	0.748-3.628	0.215			
AFP (ng/ml)	≤25/>25	1.755	0.941-3.276	0.077				1.166	0.708-1.920	0.546			
CEA (ng/mL)	≤5/>5	1.075	0.553-2.087	0.832				1.327	0.799-2.204	0.275			
CA19-9 (U/ml)	≤35/>35	1.09	0.554-2.146	0.803				0.869	0.524-1.440	0.586			
CA19-9 (U/ml)	≤200/>200	1.245	0.649-2.388	0.51				1.384	0.837-2.288	0.205			
CA19-9effect	*	1.226	0.665-2.260	0.514				1.117	0.751-1.660	0.585			
PIVKA-II(mAU/ml)	≤40/>40	2.036	0.784-5.289	0.145				1.446	0.753-2.775	0.268			
HBsAg	negative/positive	1.365	0.699-2.667	0.362				0.926	0.563-1.523	0.761			

*CA199 negative before treatment, declining after treatment if positive before treatment and no declining after treatment if positive before treatment.

### Comparisons of Complications After Treatment

The two groups of patients of two groups were evaluated for complications. There was no complication-related mortality for all included patients. The most common complications were nausea, vomiting, transient fever, abdominal pain and myelosuppression, which were controlled with symptomatic treatments. The complication rates of myelosuppression (P = 0.007) and vomiting (P = 0.006) were greater for patients in the HAIC group than those in the TACE group (**Table 3**).

**Table 3:** Comparisons of complications between two groups.

Complications	TAI	TACE	P
AII	26	9	
Myelosuppression	14	5	0.007
Vomite	6	0	0.006
fever	3	0	0.054
abdominal pain	3	4	0.896

**Figure 3:**
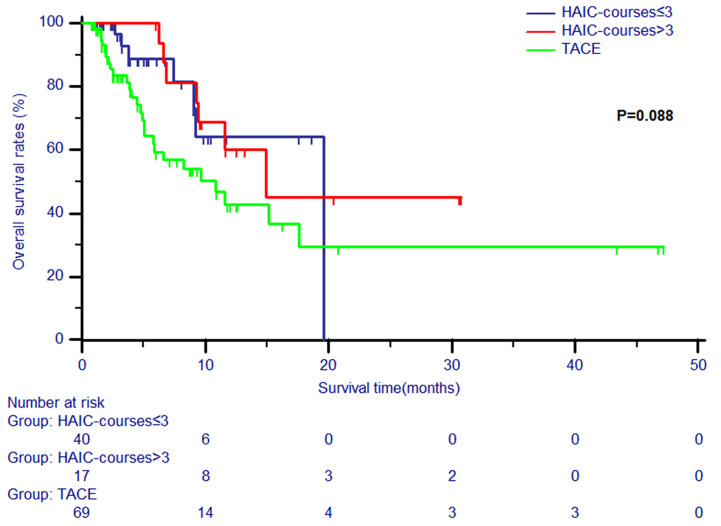
The Kaplan-Meier survival curves of overall survival stratified by two subgroups (courses of treatment > 3 and courses of treatment ≤ 3) and TACE for patients with unresectable ICC.

Patients in the HAIC group were divided into two subgroups (courses of treatment > 3 and courses of treatment ≤ 3). The survival analyses of patients in these two HAIC subgroups and TACE group were conducted. All clinical variables were balanced among these three groups (**Table 4**).It was shown that no significant differences in OS were observed in patients between the TACE group and the two HAIC subgroups (P = 0.088, **Figure 3**).

**Table 4:** Comparisons of clinical characteristics of patients.

Characteristic	HAIC-courses≤3(n = 40)	HAIC-courses>3(n = 17)	TACE(n = 69)	P
gender				
male	28	14	53	0.564
female	12	3	16	
age				
≤60	29	13	48	0.839
>60	11	4	21	
Tumor size				
≤5	4	1	5	0.829
>5	36	16	64	
Vascular Invasion				
Absence	18	10	34	0.634
Presence	22	7	35	
LN metastasis				
Absence	16	9	22	0.251
Presence	24	8	47	
Distant metastasis				
Absence	24	13	43	0.476
Presence	16	4	26	
TNM stage				
II	13	9	15	0.12
IIIb	11	4	27	
IV	16	4	27	
WBC (E+09)				
≤10	28	12	50	0.96
>10	12	5	19	
HGB (g/L)				
≤120	11	3	18	0.723
>120	29	14	51	
PLT (E+09)				
≤300	30	15	56	0.494
>300	10	2	13	
ALB (g/L)				
≤40	20	4	34	0.135
>40	20	13	35	
ALT (U/L)				
≤50	32	11	56	0.322
>50	8	6	13	
AST (U/L)				
≤40	22	5	39	0.124
>40	18	12	30	
ALP (U/L)				
≤125	16	8	30	0.875
>125	24	9	39	
GGT (U/L)				
≤60	4	2	12	0.54
>60	36	15	57	
TBIL (umol/L)				
≤20.5	35	15	55	0.485
>20.5	5	2	14	
IBIL (umol/L)				
≤15	37	17	64	0.513
>15	3	0	5	
CRP (ng/L)				
≤3	4	3	7	0.654
>3	36	14	62	
AFP (ng/ml)				
≤25	25	13	42	0.483
>25	15	4	27	
CEA (ng/mL)				
≤5	21	13	46	0.147
>5	19	4	22	
CA19-9 (U/ml)				
≤35	12	5	24	0.809
>35	28	12	44	
HBsAg				
negative	17	7	30	0.974
positive	23	9	37	
CA19-9 (U/ml)				
≤200	24	14	40	0.171
>200	16	3	29	
CA19-9effect				
Negative before operation	10	5	14	0.952
Decline after operation	16	8	15	
No decline after operation	7	4	8	
PIVKA-II(mAU/ml)				
≤40	23	11	20	0.269
>40	15	2	12	

## Discussion

Nearly half (40%-60%) of the patients with ICC are unable to undergo surgery because of advanced diseases in ICC, which is a fatal and highly malignant gastrointestinal tumor (4). Patients with unresectable ICC usually receive palliative treatment to control local tumor growth and improve the quality of life. Existing common palliative therapeutic methods include systemic chemotherapy, radiofrequency ablation (RFA), 90Y-radioembolization (RE), high dose rate brachytherapy (HDR-BT), and TACE (20–23). There are several controversies regarding treatments for unresectable ICC. In recent years, the gemcitabine/cisplatin has become the standard first-line chemotherapy regimen. Valle et al. showed that the median OS of patients in the cisplatin-gemcitabine group was 11.7 months, compared to 8.1 months of patients in the gemcitabine group; the median PFS was 8.0 months patients in the cisplatin-gemcitabine group, compared to 5.0 months in the gemcitabine group (8). This implied that FOLFOX therapy could become a promising, well-tolerated and feasible chemotherapy regimen for patients with advanced BTC (24). TACE involves the use of a combination of chemotherapy drugs and an iodized oil, which reduces the arterial supply and decreases the inflow of chemotherapeutic agents into the systemic circulation, prolonging the contact time between the cancer cells and chemotherapeutic agents and leading to a 10-25 times higher drug concentration (25). The advantage of TACE over chemotherapy has been reported by Guido Poggi et al. (26). They showed that the median OS in patients with unresectable ICC treated with OEM-TACE was 30 months, compared to 12.7 months of OS for patients in the chemotherapy group.

However, the therapeutic effect of these methods was still unsatisfactory and limited. A pilot study launched by Marumoto indicated that HAIC with CDDP, 5-FU and isovorin combined with systemic gemcitabine (GEM) may be an effective therapy for patients with advanced ICC (27). The combination therapy of PEG-IFNα-2b and 5-FU for advanced ICC achieved a median survival time of 14.6 months (14). The results of a phase II clinical trial in Cercek’s study showed the median OS of ICC patients received HAIC with FUDR was 25.0 months and the 1-year OS rate was 89.5% (28). In Jarnagin’s study (29), the median survival of ICC patients with Treatment with HAI floxuridine and systemic gemcitabine and oxaliplatin was 29.5 months and 2-year survival was 67%. There were no patients with distant metastases in these two studies. Similarly, the inclusion of patients with metastases contributed to the little inferior survival in the present study, compared with that in the Cercek’s and arnagin’s studies. Furthermore, compared with ICC patients with a median survival of 15.4 months in the ABC-trial study (30), the mFOLFOX regimen used in HAIC was shown to be a new choose for prolonging survival in ICC patients.

A prospective non-randomized study demonstrated that HAIC with mFOLFOX achieved significantly better treatment effects and had lower toxicity compared to TACE for patients with massive unresectable hepatocellular carcinoma (31). Thus, HAIC with FOLFOX might represent a feasible and promising treatment for patients with unresectable ICC. Currently, the available research results on HAIC treatment for unresectable ICC are insufficient.

In our study, the clinical response and survival differences after TACE or HAIC treatment in patients with unresectable ICC were compared. It was demonstrated that patients in the HAIC group had significantly longer OS time than patients in the TACE group, and HAIC courses were not directly relevant to the OS. Most patients tolerated these procedures well, and no patients died directly due to complications related to HAIC. Although myelosuppression and vomiting were common complications for patients treated with HAIC, most patients were able to continue the procedure after the corresponding treatment. HAIC, which differs from TACE, provides stable and sustained local delivery of chemotherapy drugs (31) and is less toxic to the surrounding liver issue (32). The primary cause of mortality is liver failure owing to the progression of intrahepatic tumors. Although there was no obvious difference in PFS between the HAIC group and the TACE group, the impact of extrahepatic metastases on survival was limited (33). Another finding from our study was that patients in the HAIC group had significantly longer OIPFS times than patients in the TACE group. Our main palliative goal was to control intrahepatic tumors in order to preserve liver function rather than achieve tumor regression. Compared to TACE, HAIC could better control the intrahepatic tumor. To avoid missing the best therapeutic opportunity, a new tumor response evaluation procedure for HAIC treatment was needed. A small minority of patients in our study presented some complications, including nausea, vomiting, transient fever, abdominal pain and myelosuppression for intrahepatic chemotherapy, but the ratio of complications in TACE or HAIC was lower than that of systemic chemotherapy, and these complications were controlled with symptomatic treatments. Therefore, HAIC may be an effective and safe therapeutic option for unresectable ICC.

There are several limitations to this study. The main defect is that a prospective, large-sample, randomized comparison was not completed. Also, our data were drawn from a single center. Some biases could not be avoided as a result of these limitations.

In conclusion, HAIC with mFOLFOX may be an effective and safe therapeutic option for unresectable ICC as an independent risk factor for prognosis. HAIC was more helpful for prolonging survival in ICC patients, compared with TACE. A new tumor response evaluation procedure for HAIC treatment is needed in order to for provide better therapeutic strategies. The results need to be confirmed by a multicenter prospective clinical study with a larger sample size.

## Data Availability Statement

The raw data supporting the conclusions of this article will be made available by the authors, without undue reservation.

## Ethics Statement

This study was approved by the Institutional Review Board of Sun Yat-sen University Cancer Center. The patients/participants provided their written informed consent to participate in this study.

## Author Contributions

ZC and CH contributed to this work equally. XL was responsible for conception, design, and quality control of this study. ZC, CH, and CZ performed the study selection, data extraction, statistical analyses, and was major contributors in writing the manuscript. ZC and CH contributed to the writing of manuscript. XL reviewed and edited the manuscript respectively. All authors contributed to the article and approved the submitted version.

## Funding

This study was supported by the grant from Guangdong Basic and Applied Basic Research Foundation (2020A1515110954) and Sun Yat-sen University Grant for Medical Humanities Practice and Teaching (No. 23000-18008023). The funding bodies did not have any influence on the design of the study, collection, analysis, interpretation of data or in writing the manuscript.

## Conflict of Interest

The authors declare that the research was conducted in the absence of any commercial or financial relationships that could be construed as a potential conflict of interest.
